# Transcriptional adaptation in *Caenorhabditis elegans*

**DOI:** 10.7554/eLife.50014

**Published:** 2020-01-17

**Authors:** Vahan Serobyan, Zacharias Kontarakis, Mohamed A El-Brolosy, Jordan M Welker, Oleg Tolstenkov, Amr M Saadeldein, Nicholas Retzer, Alexander Gottschalk, Ann M Wehman, Didier YR Stainier

**Affiliations:** 1 Department of Developmental GeneticsMax Planck Institute for Heart and Lung ResearchBad NauheimGermany; 2 Institute for Biophysical ChemistryGoethe UniversityFrankfurt Am MainGermany; 3 Cluster of Excellence Frankfurt - Macromolecular Complexes (CEF-MC)Goethe UniversityFrankfurt Am MainGermany; 4 Buchmann Institute for Molecular Life Sciences (BMLS)Goethe UniversityFrankfurt Am MainGermany; 5 Rudolf Virchow CenterUniversity of WürzburgWürzburgGermany; Howard Hughes Medical Institute, Columbia UniversityUnited States; Weill Cornell MedicineUnited States

**Keywords:** transcriptional adaptation, mRNA decay, small RNA, *C. elegans*, RNAi, *C. elegans*

## Abstract

Transcriptional adaptation is a recently described phenomenon by which a mutation in one gene leads to the transcriptional modulation of related genes, termed adapting genes. At the molecular level, it has been proposed that the mutant mRNA, rather than the loss of protein function, activates this response. While several examples of transcriptional adaptation have been reported in zebrafish embryos and in mouse cell lines, it is not known whether this phenomenon is observed across metazoans. Here we report transcriptional adaptation in *C. elegans*, and find that this process requires factors involved in mutant mRNA decay, as in zebrafish and mouse. We further uncover a requirement for Argonaute proteins and Dicer, factors involved in small RNA maturation and transport into the nucleus. Altogether, these results provide evidence for transcriptional adaptation in *C. elegans*, a powerful model to further investigate underlying molecular mechanisms.

## Introduction

Transcriptional adaptation is the ability of certain mutations in a gene to modulate the expression of related genes, referred to as adapting genes (El-Brolosy and Stainier, 2017; El-Brolosy et al., 2019; Ma et al., 2019). At the molecular level, the mutant mRNA, rather than the loss of protein function, is responsible for this transcriptional modulation (Rossi et al., 2015; El-Brolosy and Stainier, 2017; Sztal et al., 2018; El-Brolosy et al., 2019; Ma et al., 2019). According to one model (El-Brolosy et al., 2019), the mutant mRNA, via its degradation products, modulates the expression of adapting genes via transcriptional regulators including antisense RNAs and histone modifiers. According to another model (Ma et al., 2019), the premature termination codon (PTC) containing mutant mRNA interacts with a histone modifier complex leading to transcriptional upregulation of the adapting gene(s). Sequence similarity with the mutant mRNA determines which genes get upregulated during transcriptional adaptation (El-Brolosy et al., 2019). In some cases, the upregulated genes share functionality with the mutated gene leading to functional compensation. However, while transcriptional adaptation is often discussed in the context of genetic robustness (Rossi et al., 2015; El-Brolosy et al., 2019; Ma et al., 2019), it does not always lead to functional compensation (Rossi et al., 2015). In addition, transcriptome analyses suggest that even genes with limited sequence similarity with the mutant mRNA can be upregulated during transcriptional adaptation (El-Brolosy et al., 2019), although clearly more work is required to determine whether the upregulation of these genes is a direct or indirect effect of transcriptional adaptation.

Understanding the mechanisms of transcriptional adaptation will help us better comprehend why for a given gene some mutations cause disease while others do not (Castel et al., 2018). However, despite the importance and growing interest in many aspects of genetic compensation, transcriptional adaptation has currently only been investigated in vertebrates. Thus, it remains unclear whether this phenomenon is observed across metazoans. The evolutionary importance of related genes that have compensatory effects has also been discussed in non-vertebrate eukaryotes (Conant and Wagner, 2004; Plata and Vitkup, 2014). However, it is not known whether these examples of compensation are due to protein feedback loops or transcriptional adaptation.

Only a few factors are known to be involved in the transcriptional adaptation response thus far, and others, including some involved in RNA processing and transport, are likely required. In addition, it is not clear whether the mechanisms of transcriptional adaptation are common or whether each particular case occurs in a different manner, especially at the step leading to transcriptional modulation. Also, as different paralogs or related genes are expressed in distinct tissues and/or at different times (Laisney et al., 2010; Kryuchkova-Mostacci and Robinson-Rechavi, 2015; Radomska et al., 2016; Pasquier et al., 2017; Jojic et al., 2018), it is currently unclear whether the expression of adapting genes can appear in tissues where, and/or at times when, they are not normally expressed.

In this study, we provide examples of transcriptional adaptation in *Caenorhabditis elegans* and show the ectopic expression of an extrachromosomal reporter in a tissue where it is not normally expressed. In addition, we analyze these transcriptional adaptation models after RNAi-mediated knockdown of different genes involved in RNA metabolism and find that the upregulation of the adapting genes requires factors involved in the maturation and transport into the nucleus of small RNAs (sRNAs).

## Results

### Examples of transcriptional adaptation in *C. elegans*

**Figure 1.**
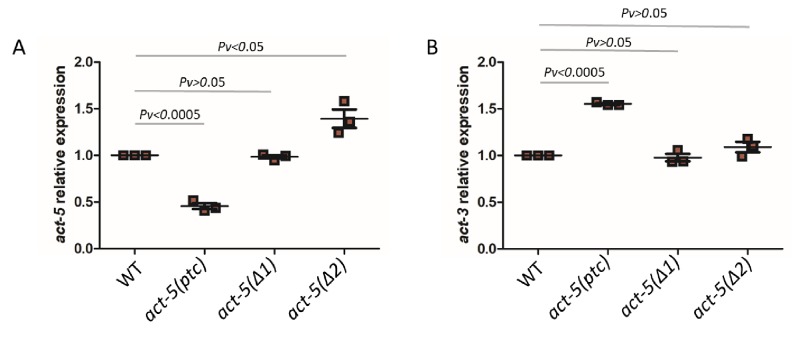
mRNA levels of *act-5* and *act-3* in WT and mutant alleles. qPCR analysis of *act-5* (**A**) and *act-3* (**B**) mRNA levels in WT and *act-5(ptc)*, *act-5(Δ1)*, and *act-5(Δ2)* mutants. *act-3* mRNA levels are upregulated when *act-5* mutant mRNA levels are reduced (i.e., only in the *act-5(ptc)* allele). WT expression levels are set at 1. Data are mean ± S.E.M.; average dCt values are shown in Figure 1—source data 1. Two-tailed Student’s t-test was used to calculate *P* values. Figure 1—source data 1.Average dCt values from qPCR analysis of *act-5* and *act-3* mRNA levels in WT and *act-5* mutants. Figure 1—source data 2.Average dCt values from qPCR analysis of *act-1*, *act-2* and *act-4* mRNA levels in WT and *act-5(ptc)* mutants.

Actins are essential structural components of eukaryotic cells as they mediate a wide range of cellular processes (Pollard and Cooper, 1986). Actin genes are often present in multiple copies in higher eukaryotic genomes and hints of transcriptional adaptation modulating their expression have been reported. For example, mouse embryonic fibroblasts (MEFs) mutant for *β-Actin* (*Actb*) display increased expression of other Actins including α- and γ-Actin (ACTA and ACTG1/2) as measured by Western blots (Tondeleir et al., 2012). Similarly, *Actg1* knockout, but not knockdown, in MEFs leads to an increase in *Acta* mRNA levels (Patrinostro et al., 2017), and zebrafish *actc1b* mutants exhibit mild muscle defects because of the transcriptional upregulation of *actc1a* (Sztal et al., 2018). Furthermore, *Actg1* mutant MEFs and *Actb* mutant mouse embryonic stem cells (mESCs) display increased mRNA levels of *Actg2* and *Actg1*, respectively, and this upregulation is triggered not by the loss of protein function but by mutant mRNA decay (El-Brolosy et al., 2019). Thus, we decided to investigate actin genes in *C. elegans* to test for transcriptional adaptation. The *C. elegans* genome contains five actin genes which display high similarity in their DNA and protein sequences (MacQueen et al., 2005). We started by analyzing several mutant alleles for *act-1*, *act-2*, *act-3* and *act-5*, and determined mutant transcript levels. We found significantly reduced *act-5* mRNA levels in *act-5(dt2019)* mutants compared to wild type (Figure 1A), likely caused by nonsense-mediated decay (NMD) due to a premature termination codon (ptc) in the first exon (Figure 1A, Figure 1—figure supplement 1). Most mutant alleles of *act-5* cause severe phenotypes including lethality (Estes et al., 2011; MacQueen et al., 2005), sterility (Cui et al., 2004), and paralysis (Etheridge et al., 2015). However, the *act-5(dt2019)* allele, hereafter referred to as *act-5(ptc)*, does not exhibit any obvious phenotype (MacQueen et al., 2005), an observation we confirmed. We analyzed the mRNA levels of all actin genes in *act-5(ptc)* mutants (Figure 1A, Figure 1—figure supplement 2), and observed the upregulation of *act-3* mRNA and pre-mRNA (Figure 1B, Figure 1—figure supplement 3), consistent with a transcriptional adaptation response.

**Figure 1—figure supplement 1.**
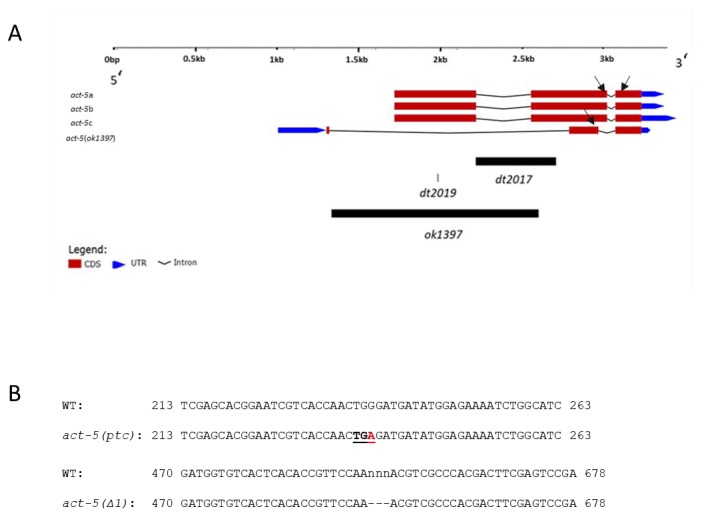
Organization of *act-5* locus. (**A**) Schematic of all known *act-5* isoforms and alleles used in this study. Black boxes indicate the deletion and nonsense alleles used in this study; black arrows point to the position of the qPCR primers. The *act-5(ok1397)* isoform was only identified in the *act-5(Δ2)* allele. (**B**) Partial sequence of *act-5* (WT, *ptc* and *Δ1* alleles). The PTC in the *act-5(ptc)* allele is 264 nucleotides from the next exon-intron junction and 888 nucleotides from the stop codon. Red indicates the mutation; PTC is underlined; ‘nnn’ and ‘---’ indicate deleted nucleotides in the *act-5(Δ1)* allele. The *dt2017* deletion leads to a PTC which is located in the last exon (153 bases from the stop codon) and is thus predicted not to trigger NMD (Kashima et al., 2010; Lindeboom et al., 2016). *act-5(dt2019)* = *act-5(ptc)*; *act-5(dt2017)* = *act-5(Δ1)*; *act-5(ok1397)* = *act-5(Δ2)*.

**Figure 1—figure supplement 2.**
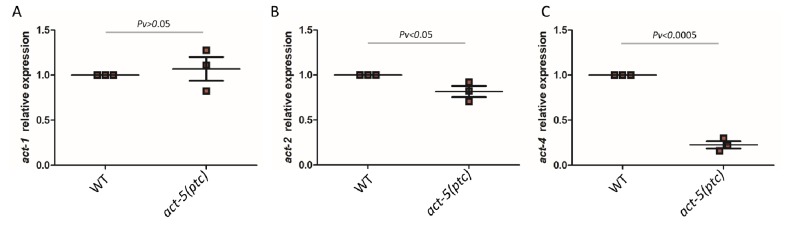
mRNA levels of *act-1* (**A**), *act-2* (**B**) and *act-4* (**C**) in WT and *act-5(ptc)* mutants. WT expression levels are set at 1. Data are mean ± S.E.M.; average dCt values are shown in Figure 1—source data 2. Two-tailed Student’s t-test was used to calculate *P* values.

**Figure 1—figure supplement 3.**
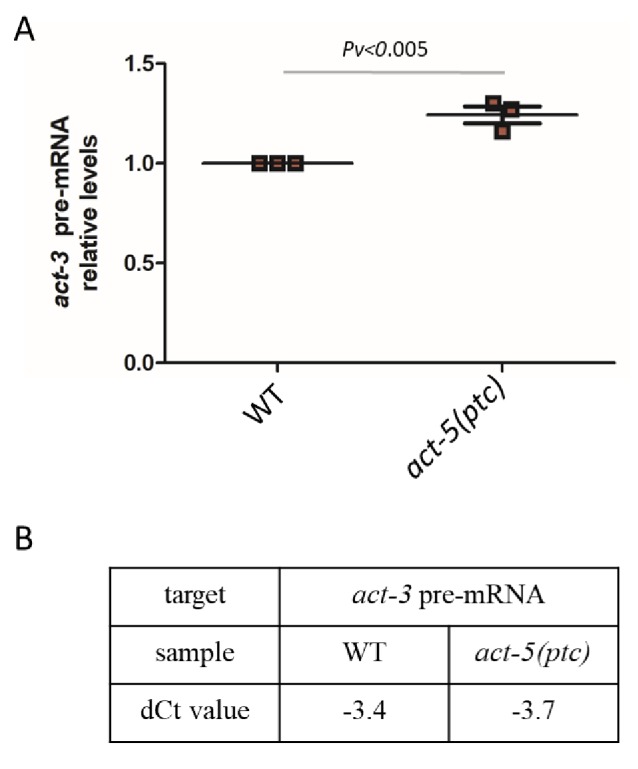
Pre-mRNA levels of *act-3* in WT and *act-5(ptc)* mutants. (**A**) qPCR analysis of *act-3* pre-mRNA levels in WT and *act-5(ptc)* mutants. (**B**) Average dCt values from qPCR analysis of *act-3* pre-mRNA levels in WT and *act-5(ptc)* mutants. WT expression levels are set at 1. Data are mean ± S.E.M.. Two-tailed Student’s t-test was used to calculate *P* values.

We also examined the *act-5*(*dt2017*) partial deletion allele, hereafter referred to as *act-5(Δ1)*, (Figure 1—figure supplement 1) and found no significant change in *act-5* mRNA levels in homozygous mutants compared to wild type (Figure 1A). Notably, *act-3* mRNA levels in *act-5(Δ1)* mutants were not changed compared to wild type (Figure 1B). To further test whether *act-3* upregulation in *act-5* mutants represents a model of transcriptional adaptation, we analyzed another *act-5* deletion allele (*ok1397*) (Estes et al., 2011), hereafter referred to as *act-5(Δ2)*. The *ok1397* deletion removes part of the promoter region and the first two exons (Figure 1—figure supplement 1). We examined this allele for the presence of any transcripts and identified a new isoform which is present in mutants but not in wild type (see Materials and methods) and consists of only 3’ sequence (Figure 1—figure supplement 1). As with the *act-5*(*Δ1*) deletion allele, we found no changes in *act-5* or *act-3* mRNA levels in *act-5(Δ2)* mutants compared to wild type (Figure 1B).

**Figure 2.**
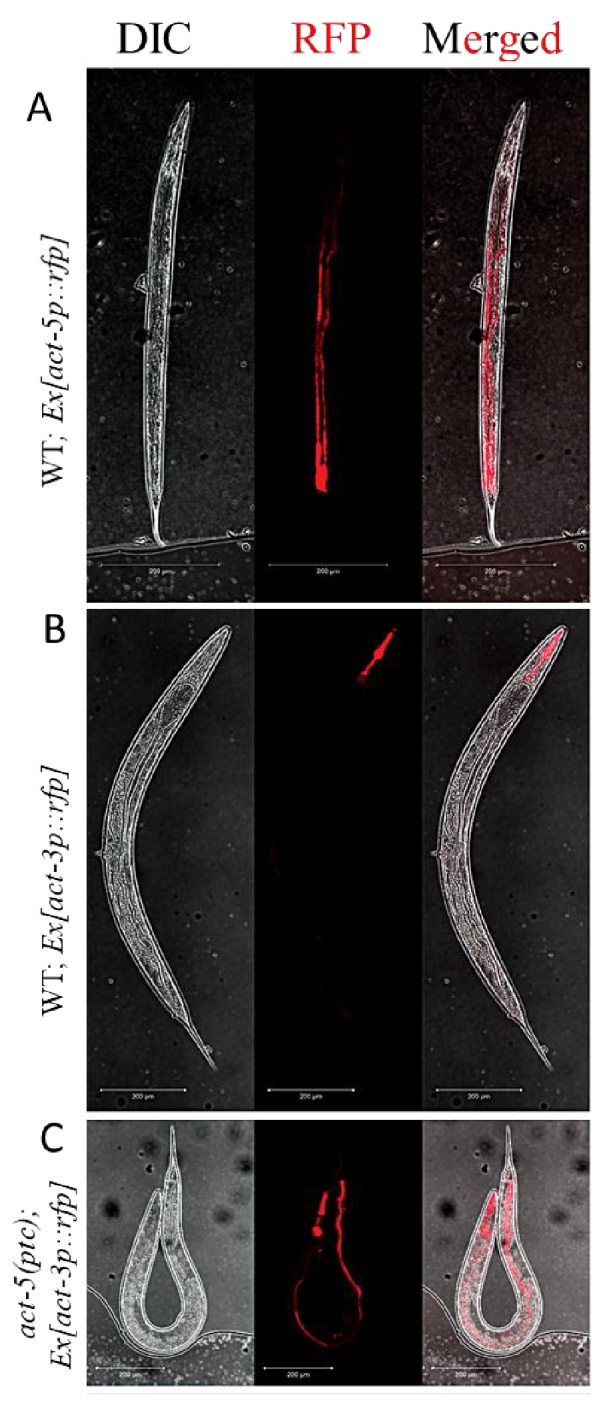
Extrachromosomal reporter expression in WT and mutant alleles. (**A**) *act-5p::rfp* extrachromosomal reporter expression was observed in the intestine in 153 of 300 WT animals. (**B**) *act-3p::rfp* extrachromosomal reporter expression was observed in the pharynx in 182 of 400 WT animals. (**C**) *act-3p::rfp* extrachromosomal reporter expression was observed in the pharynx and intestine in 138 of 320 *act-5(ptc)* mutants.

In multicellular organisms, paralogous genes are often expressed in distinct spatiotemporal patterns, an indication of subfunctionalization (Guschanski et al., 2017). For example, in *C. elegans*, *act-3* is expressed in the pharynx (Hunt-Newbury et al., 2007) while *act-5* is expressed in intestinal cells (MacQueen et al., 2005). The models of transcriptional adaptation suggest a cell-autonomous mechanism, that is the mutant mRNA can cell-autonomously trigger transcriptional adaptation. In order to test this hypothesis, we generated transcriptional reporter constructs with the *act-3* or *act-5* promoter region driving the expression of a red florescent protein gene (Merzlyak et al., 2007). As expected, we observed expression of the extrachromosomal *act-5p::rfp* transgene in the intestine in wild-type animals (Figure 2A) as well as in *act-5(ptc)* mutants (Figure 2—figure supplement 1). Likewise, expression of the extrachromosomal *act-3p::rfp* transgene was only observed in the pharynx in wild-type animals (Figure 2B), consistent with previous studies (Hunt-Newbury et al., 2007). However, extrachromosomal *act-3p::rfp* expression was also observed in the intestine in *act-5(ptc)* mutants (Figure 2C), consistent with transcriptional adaptation. In summary, we saw upregulation of expression from a synthetic and extrachromosomal *act-3* promoter in tissues where *act-5* is expressed, supporting the model that the mutant mRNA cell-autonomously triggers transcriptional adaptation.

**Figure 2—figure supplement 1.**
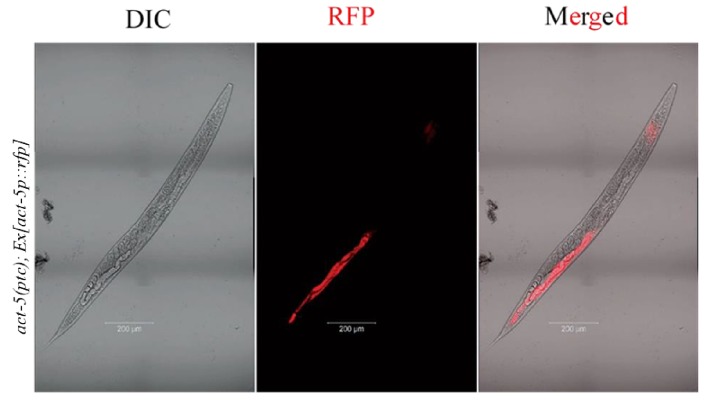
*act-5p::rfp* extrachromosomal reporter expression. *act-5p::rfp* extrachromosomal reporter expression was observed in the pharynx and intestine in 148 of 300 *act-5(ptc)* mutants.

**Figure 3.**
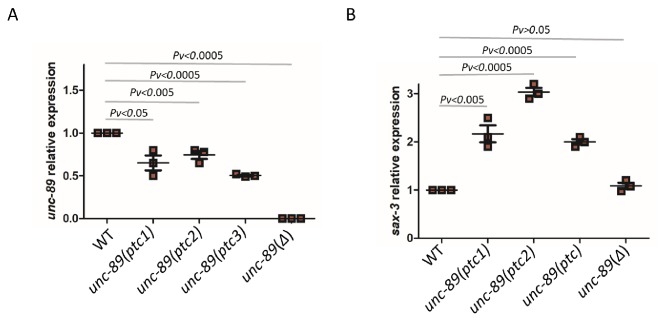
mRNA levels of *unc-89* and *sax-3* in WT and mutant alleles. qPCR analysis of *unc-89* (**C**) and *sax-3* (**D**) mRNA levels in WT and *unc-89(ptc1)*, *unc-89(ptc2)*, *unc-89(ptc3)*, and *unc-89(Δ)* mutants. *sax-3* mRNA levels in *unc-89* alleles are upregulated when *unc-89* mutant mRNA levels are reduced, except in the deletion allele. WT expression levels are set at 1. Data are mean ± S.E.M.; average dCt values are shown in Figure 3—source data 2. Two-tailed Student’s t-test was used to calculate *P* values. Figure 3—source data 1.List of *ttn-1* paralogous genes based on WormBase release WS266. Figure 3—source data 2.Average dCt values from qPCR analysis of *unc-89* and *sax-3* mRNA levels in WT and *unc-89* mutants. Figure 3—source data 3.Distance, in nucleotides, from each PTC to the next exon-intron junction and to the stop codon in each *unc-89* isoform in the *unc-89(ptc1)*, *unc-89(ptc2)*, and *unc-89(ptc3)* alleles.‘-’ indicates the absence of the PTC from the isoform.

To identify an additional example of transcriptional adaptation in *C. elegans*, we turned to the *titin* gene family (Figure 3—source data 1). Due to their size, *titin* genes are frequent targets of random mutagenesis, and several PTC alleles have been identified (Jorgensen and Mango, 2002; Lipinski et al., 2011). We focused on *unc-89* which has many nonsense alleles that do not exhibit an obvious phenotype, potentially indicating functional compensation. We identified three different *unc-89* alleles (*gk469156*, *gk509355*, *gk506355*) which exhibit lower levels of mutant mRNA compared to wild type (Figure 3A) and lack an obvious phenotype. Analyzing the mRNA levels of 10 *titin* related genes (*him-4*, *ttn-1*, *ketn-1*, *sax-3*, *unc-22*, *unc-52*, *sax-7*, *rig-6*, *unc-40*, and *unc-73*), we found that *sax-3* was upregulated in all three *unc-89*(*ptc*) alleles (Figure 3—figure supplement 1), both at the mRNA (Figure 3B) and pre-mRNA (Figure 3—figure supplement 2) levels. To test whether this upregulation of *sax-3* was due to transcriptional adaptation and not to the loss of UNC-89 function, we generated a 16 kb deletion (*bns7000*) in *unc-89*, hereafter referred to as *unc-89*(*Δ*), using CRISPR/Cas9 genome editing. This deletion removes part of the promoter region and the first several exons (Figure 3—figure supplement 1). Hence, most *unc-89* isoforms are not observed in *unc-89(Δ)* mutants (Figure 3A). Homozygous *unc-89(Δ)* worms are maternal-effect sterile and exhibit growth defects, phenotypes not observed in the *unc-89(ptc)* alleles. In the RNA-less *unc-89(Δ)* allele, *sax-3* was not upregulated (Figure 3B), indicating that *sax-3* upregulation is not due to the loss of UNC-89 function and that the mutant mRNA needs to be present for the transcriptional adaptation response. Thus, *sax-3* upregulation in the *unc-89(ptc)* alleles is a second example of transcriptional adaptation in *C. elegans*.

**Figure 3—figure supplement 1.**
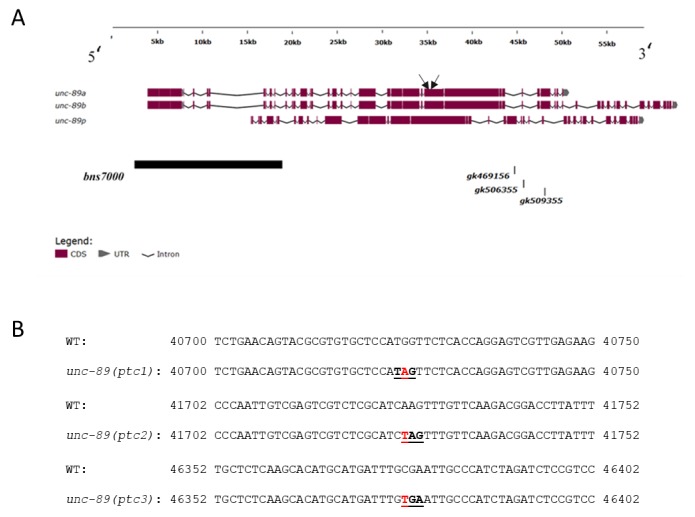
Organization of *unc-89* locus. Schematic of a subset of the 16 known *unc-89* isoforms as well as the deletion and nonsense alleles used in this study (black boxes). Black arrows point to the position of the qPCR primers. (**B**) Partial sequence of the longest *unc-89* isoform with the single nucleotide change causing PTCs. Red indicates the mutations; PTCs are underlined. The distance from each PTC to the next exon-intron junction and to the stop codon is shown in Figure 3—source data 3. *unc-89* (*gk469156*)* = unc-89(ptc1); unc-89* (*gk506355*)* = unc-89(ptc2); unc-89* (*gk509355*)* = unc-89(ptc3); unc-89(bns7000*)* = unc-89(Δ*).

**Figure 3—figure supplement 2.**
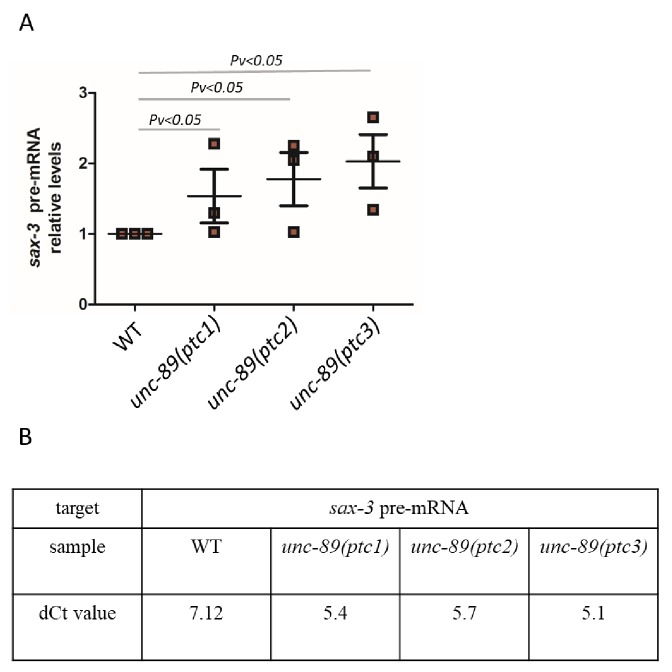
Pre-mRNA levels of *sax-3* in WT and *unc-89(ptc1)*, *unc-89(ptc2)*, *unc-89(ptc3)* mutants. (**A**) qPCR analysis of *sax-3* pre-mRNA levels in WT and *unc-89(ptc1)*, *unc-89(ptc2)*, *unc-89(ptc3)* mutants. (**B**) Average dCt values from qPCR analysis of *sax-3* pre-mRNA levels in WT and *unc-89(ptc1)*, *unc-89(ptc2)*, *unc-89(ptc3)* mutants. WT expression levels are set at 1. Data are mean ± S.E.M.. Two-tailed Student’s t-test was used to calculate *P* values.

**Figure 3—figure supplement 3.**
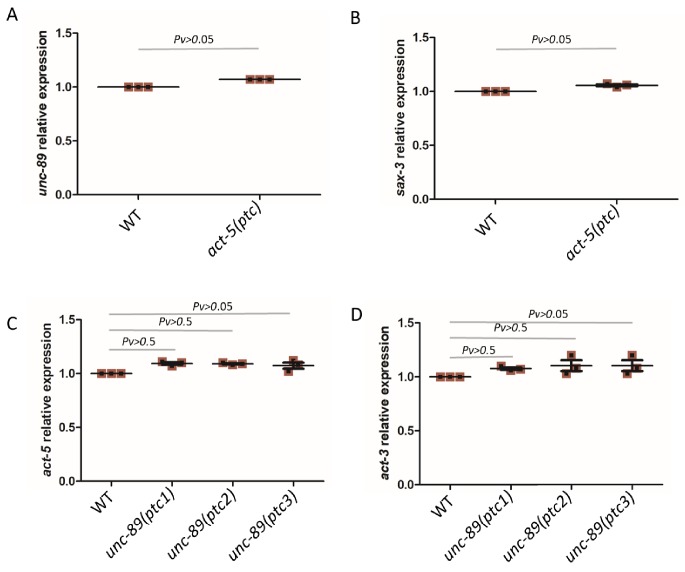
mRNA levels in WT and mutant alleles. qPCR analysis of *unc-89* (**A**) and *sax-3* (**B**) mRNA levels in WT and *act-5(ptc)* mutants as well as of *act-5* (**C**) and *act-3* (**D**) in WT and *unc-89(ptc1)*, *unc-89(ptc2) and unc-89(ptc3)* mutants. WT expression levels are set at 1. Data are mean ± S.E.M.; Two-tailed Student’s t-test was used to calculate *P* values.

To test whether the observed changes in gene expression in *act-5(ptc)* and *unc-89(ptc)* mutants were specific, we measured *unc-89* and *sax-3* expression in *act-5(ptc)* mutants as well as *act-5* and *act-3* expression in *unc-89(ptc)* mutants. We observed no significant differences (Figure 3—figure supplement 3), suggesting that there is specificity to the gene expression changes.

### Identifying additional regulators of transcriptional adaptation

The mutant mRNA has been reported to activate transcriptional adaptation in zebrafish embryos and mouse cell lines (El-Brolosy et al., 2019; Ma et al., 2019). In order to identify additional factors involved in transcriptional adaptation, we performed a candidate RNA interference (RNAi) screen, focusing on genes involved in RNA metabolism (Figure 4—source data 1). We knocked down genes involved in mRNA processing including splicing and nonsense-mediated decay, as well as other genes involved in small RNA synthesis and maturation. We measured the mRNA levels of the mutant and adapting genes in order to position RNAi candidates upstream or downstream of mRNA decay. If the gene targeted by RNAi is required for mutant mRNA decay, we expect to see the mRNA levels of the mutant and adapting genes to be similar to wild-type levels. If the gene targeted by RNAi is involved in transcriptional adaptation downstream of mutant mRNA decay, we expect to see the levels of mutant mRNA remaining lower than in wild type, but the levels of the adapting gene’s mRNA to be similar to wild-type levels. Finally, if the gene targeted by RNAi is not involved in transcriptional adaptation, we expect to see the levels of mutant mRNA remain lower than in wild type and the expression levels of the adapting gene to remain higher than in wild type. For example, when we knocked down *drsh-1*, a gene involved in miRNA biogenesis (Denli et al., 2004), we saw no significant changes in the mRNA levels of the mutant or adapting genes compared to control (Figure 4—figure supplement 1A,B,C,D), suggesting that *drsh-1* is not involved in regulating transcriptional adaptation.

**Figure 4.**
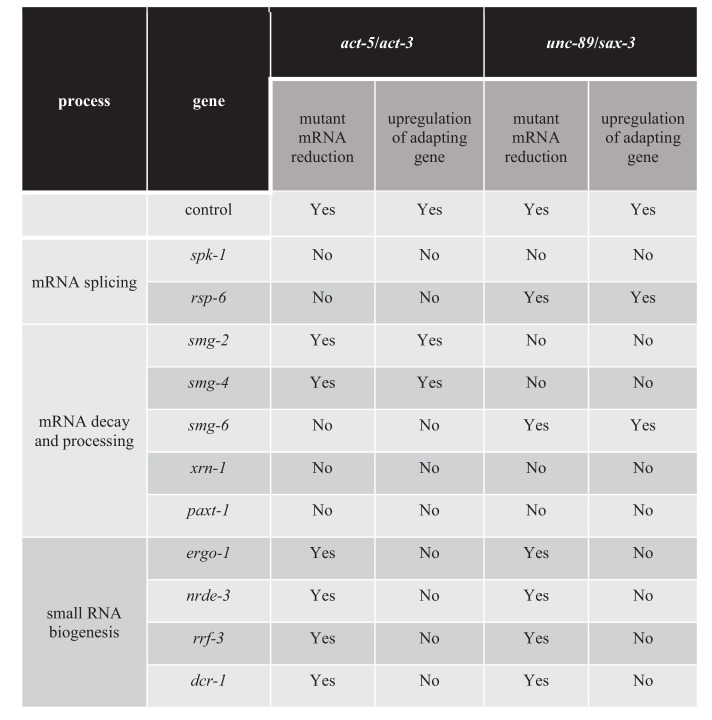
Factors regulating transcriptional adaptation identified in RNAi-mediated knockdown screen. Figure 4—source data 1.List of genes and RNAi clones tested in the screen; average dCt values of qPCR analyses of *act-5* and *act-3* mRNA levels in WT and *act-5* mutants as well as of *unc-89* and *sax-3* mRNA levels in WT and *unc-89* mutants.

Transcriptional adaptation requires the activity of decay factors (El-Brolosy et al., 2019; Ma et al., 2019), and UPF1, SMG6, and XRN1 were reported to be differentially required in various zebrafish embryo and mouse cell line models of transcriptional adaptation (El-Brolosy et al., 2019). In order to test whether NMD factors are involved in regulating transcriptional adaptation in *C. elegans*, we knocked down several NMD genes including *smg-2* (the *C. elegans* orthologue of *Upf1*), *smg-4* (*Upf3*) and *smg-6* (*Smg6*). Knockdown of *smg-2* and *smg-4* blocked the transcriptional adaptation response in all three *unc-89(ptc)* alleles but not in the *act-5(ptc)* allele (Figure 4). Conversely, knockdown of *smg-6* blocked the transcriptional adaptation response in the *act-5(ptc)* allele but not in the three *unc-89(ptc)* alleles (Figure 4). A differential requirement for Upf1 and Smg6 between gene models was also observed in mouse cells (El-Brolosy et al., 2019).

**Figure 4—figure supplement 1.**
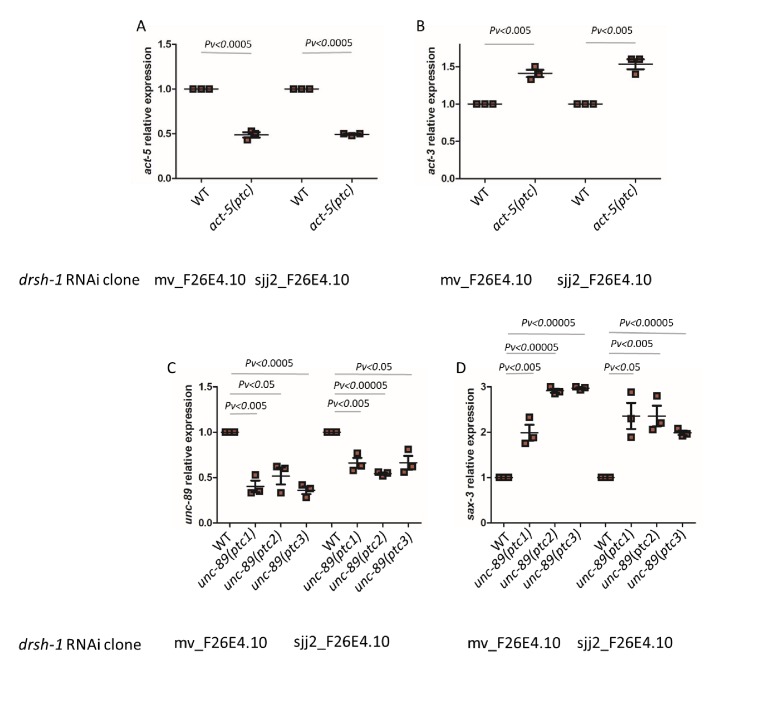
qPCR analysis of *act-5* (**A**) and *act-3* (**B**) mRNA levels in WT and *act-5(ptc)* mutants as well as of *unc-89* (**C**) and *sax-3* (**D**) mRNA levels in WT and *unc-89(ptc)* mutants upon *drsh-1* RNAi-mediated knockdown by two independent clones. WT expression levels are set at 1. Data are mean ± S.E.M.; average dCt values are shown in Figure 4—source data 1. Two-tailed Student’s t-test was used to calculate *P* values.

**Figure 4—figure supplement 2.**
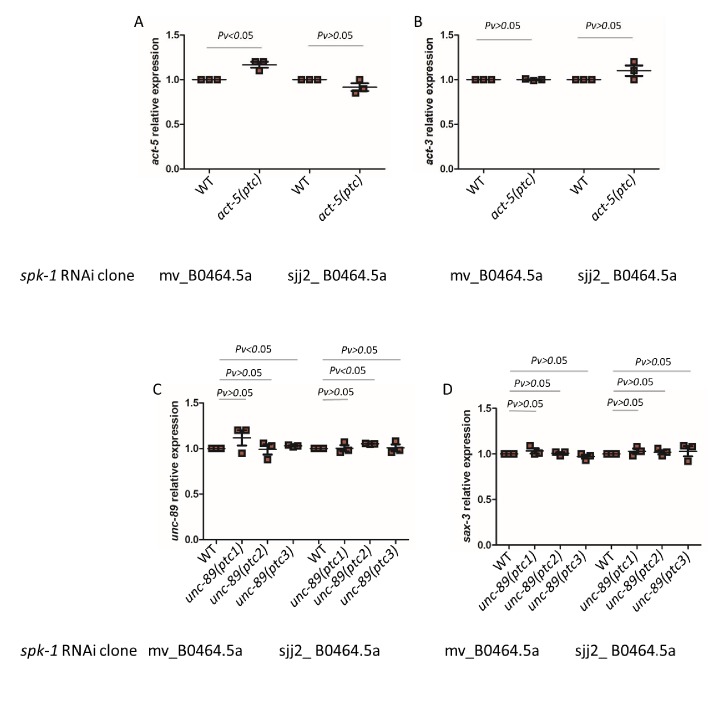
qPCR analysis of *act-5* (**A**) and *act-3* (**B**) mRNA levels in WT and *act-5(ptc)* mutants as well as of *unc-89* (**C**) and *sax-3* (**D**) mRNA levels in WT and *unc-89(ptc)* mutants upon *spk-1* RNAi-mediated knockdown by two independent clones. WT expression levels are set at 1. Data are mean ± S.E.M.; average dCt values are shown in Figure 4—source data 1. Two-tailed Student’s t-test was used to calculate *P* values.

**Figure 4—figure supplement 3.**
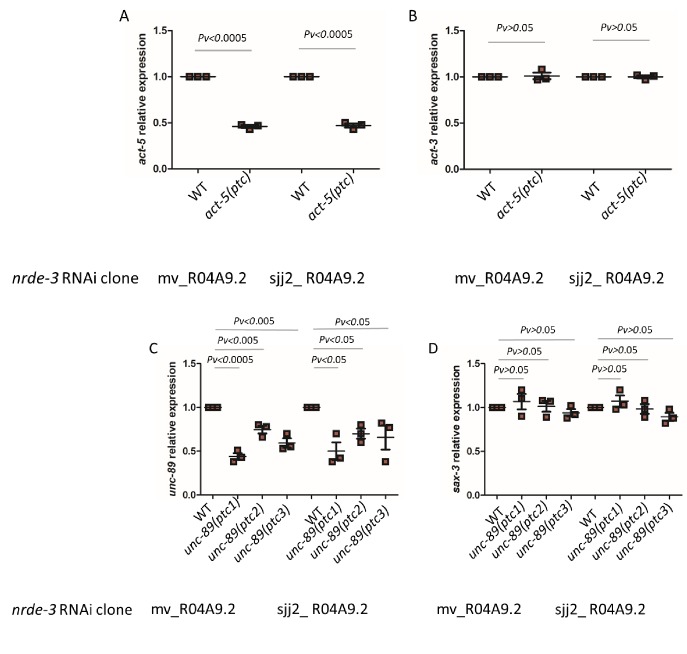
qPCR analysis of *act-5* (**A**) and *act-3* (**B**) mRNA levels in WT and *act-5(ptc)* mutants as well as of *unc-89* (**C**) and *sax-3* (**D**) mRNA levels in WT and *unc-89(ptc)* mutants upon *nrde-3* RNAi-mediated knockdown by two independent clones. WT expression levels are set at 1. Data are mean ± S.E.M.; average dCt values are shown in Figure 4—source data 1. Two-tailed Student’s t-test was used to calculate *P* values.

**Figure 5.**
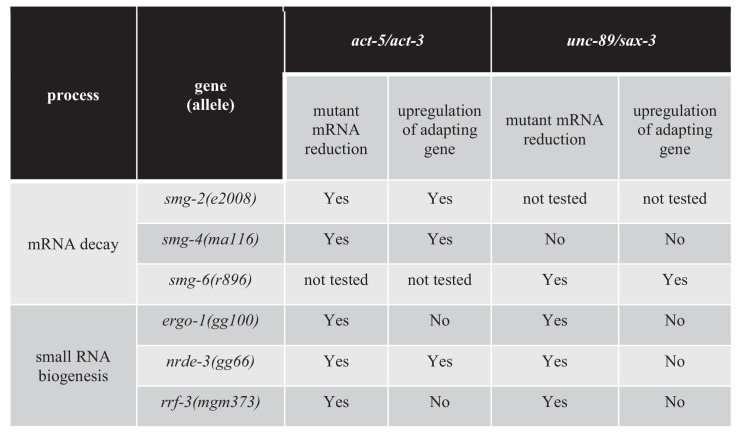
Factors regulating transcriptional adaptation analyzed in double mutants. Figure 5—source data 1.List of genes and alleles for each gene tested in the double mutant analysis; average dCt values from qPCR analyses of *act-5* and *act-3* mRNA levels in WT and *act-5* mutants as well as of *unc-89* and *sax-3* mRNA levels in WT and *unc-89* mutants.

As RNAi efficiency can vary in different tissues (Ratliff et al., 2006; Zhuang and Hunter, 2011), we generated double mutant strains with *smg-2*, *smg-4*, or *smg-6* mutant alleles and the *act-5(ptc)* and *unc-89(ptc)* alleles to exclude the possibility of tissue-specific knockdown. Analysis of the double mutant strains confirmed the observations made in the RNAi experiments (Figure 5). For example, we found that the levels of *act-5* mRNA were lower in *smg-2; act-5(ptc)* and *smg-4; act-5(ptc)* double mutants than in *smg-2* and *smg-4* single mutants, and that the levels of the adapting gene’s mRNA were higher (Figure 5—source data 1), further indicating that *smg-2* and *smg-4* are not required for transcriptional adaptation in the *act-5* model. However, in *smg-4; unc-89(ptc)* animals, the mRNA levels of the mutant (*unc-89*) and adapting (*sax-3*) genes were similar to those in *smg-4* single mutants. Furthermore, these animals exhibited a mild uncoordinated phenotype and grew slowly, suggesting a lack of functional compensation. These data further indicate that *smg-4* is required for transcriptional adaptation in the *unc-89* model. We could not obtain *smg-6; act-5(ptc)* viable mutants due to severe larval lethality, possibly as a consequence of blocking the transcriptional adaptation response, that is *act-3* upregulation. *smg-6; unc-89(ptc)* mutants exhibited lower levels of *unc-89* mRNA and higher levels of adapting gene mRNA in comparison to single *smg-6* mutants (Figure 5—source data 1) similar to the observations in the RNAi experiments. Thus, there are differential requirements for decay factors in different models of transcriptional adaptation.

**Figure 5—figure supplement 1.**
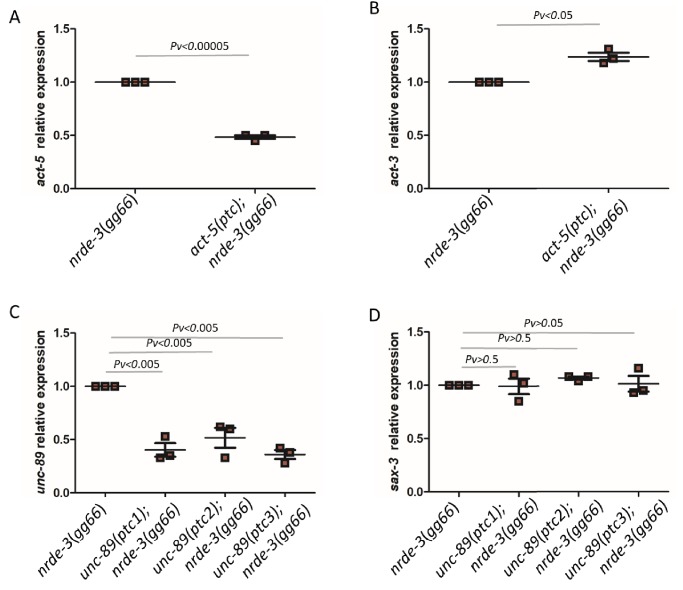
Partial data from double mutant analysis. qPCR analysis of *act-5* (**A**) and *act-3* (**B**) mRNA levels in *nrde-3(gg66)* mutants and *act-5(ptc); nrde-3(gg66)* double mutants as well as of *unc-89* (**C**) and *sax-3* (**D**) mRNA levels in *nrde-3(gg66)* and *unc-89(ptc); nrde-3(gg66)* double mutants. Single mutant *nrde-3(gg66)* expression levels are set at 1. Data are mean ± S.E.M.; average dCt values are shown in Figure 5—source data 1. Two-tailed Student’s t-test was used to calculate *P* values.

Previous data indicate that the exonuclease Xrn1 is involved in regulating the transcriptional adaptation response in mouse cells (El-Brolosy et al., 2019). Therefore, we tested the role of exonucleases in transcriptional adaptation in *C. elegans*, specifically the exonuclease gene *xrn-1* (Jones et al., 2012) and the XRN-2 partner gene *paxt-1* (Miki et al., 2014) (Figure 4). We found that knocking down *xrn-1* or *paxt-1* led to mutant (*act-5* and *unc-89*) mRNA levels similar to wild-type levels. Furthermore, the transcriptional adaptation response was blocked, suggesting that the degradation and processing of mutant transcripts is important to trigger transcriptional adaptation.

We next looked for additional factors required for transcriptional adaptation. Pre-mRNA splicing and NMD are closely related processes via the positioning and use of the exon junction complex (EJC) (Lejeune and Maquat, 2005; Kashima et al., 2010; Fukumura et al., 2016). SR-protein kinases (SRPK) and their substrates, serine/arginine-rich (SR) splicing factors, are key components of the splicing machinery and are well conserved across phyla (Kuroyanagi et al., 2000; Black, 2003; Galvin et al., 2011). Multiple SR proteins are components of the EJC (Singh et al., 2012), consistent with a previously suggested role of SR proteins in mRNA surveillance (Zhang and Krainer, 2004). Knocking down the SRPK gene *spk-1* resulted in mutant mRNA levels similar to wild-type levels, and blocked transcriptional adaptation in all *act-5* and *unc-89 ptc* alleles (Figure 4—figure supplement 2A,B,C,D). We also identified the SR family gene *rsp-6* as a regulator of transcriptional adaptation in the *act*-*5* model, but were unable to identify a single SR protein whose knockdown influenced the transcriptional adaptation response in the *unc-89* model (Figure 4—source data 1), possibly due in part to the complexity of the *unc-89* gene structure including the large number of isoforms (Tourasse et al., 2017).

The next group of genes we targeted encode factors involved in small RNA (sRNA) biogenesis, maturation and transport into the nucleus. We tested several pathways (Figure 4—source data 1) and observed that the argonaute proteins ERGO-1 and NRDE-3, the RNA-dependent RNA polymerase RRF-3, as well as the ribonuclease DCR-1 regulate the transcriptional adaptation response downstream of mRNA decay (*i.e*., the mutant mRNA was still degraded but the adapting gene was not upregulated) (Figure 4, Figure 4—figure supplement 3A,B,C,D, Figure 4—source data 1). These RNAi data were confirmed by analyzing double mutants of the *act-5(ptc)* or *unc-89(ptc)* alleles with *ergo-1*, *nrde-3*, and *rrf-3* (Figure 5), and all these animals exhibited phenotypes comparable to the *act-5* or *unc-89* deletion alleles analyzed in this study including larval lethality, slow growth and uncoordinated movements, indicating lack of functional compensation. Notably, ERGO-1, NRDE-3, RRF-3, and DCR-1 are involved in 26G RNA biogenesis (Pavelec et al., 2009; Vasale et al., 2010; Fischer, 2010; Grishok, 2013; Yvert, 2014), suggesting that 26G RNAs could play a role in transcriptional adaptation.

Together, these results indicate that mRNA decay as well as small RNA biogenesis and transport are critical in triggering transcriptional adaptation.

## Discussion

Recent advances in reverse genetic tools have significantly expanded our ability to generate genetic modifications in a wide range of organisms (Housden et al., 2017). However, some engineered mutants exhibit no apparent phenotype, renewing interest in the concept of genetic robustness. Genetic compensation, and in particular transcriptional adaptation, have been proposed as a means to achieve genetic robustness upstream of protein feedback loops. Despite the potential importance of transcriptional adaptation, its underlying molecular mechanisms remain relatively unexplored. Here, we report two cases of transcriptional adaptation in *C. elegans*. By carrying out a small RNAi screen and a follow up analysis using double mutants, we identified several new factors that regulate transcriptional adaptation and further validated previously identified ones.

In the *C. elegans act-5* model, the mutant gene and related adapting gene (*act-5* and *act-3*, respectively) are primarily expressed in distinct tissues. However, using an extrachromosomal transcriptional reporter, we observed that in *act-5(ptc)* mutants, the *act-3* promoter adapts to drive transcription in the primary site of *act-5* expression, the intestine. As *act-5(ptc)* mutants do not exhibit any obvious phenotype when the transcriptional adaptation response is intact, we predict that ACT-3 and/or other proteins are able to compensate for the loss of ACT-5. Indeed, when we disrupted transcriptional adaptation, *act-5(ptc)* mutants did not survive. Thus, transcriptional adaptation can in some cases entail the change in the pattern of expression of related gene(s) and suppress phenotypes that would alter the animal’s fitness.

Based on the factors identified in this study, we hypothesize that the transcriptional adaptation response consists of at least three critical processes: mutant mRNA decay, sRNA maturation and sRNA transport. In terms of mutant mRNA decay, we found that the machinery can be gene-specific. In our experiments, SMG-6 is involved in *act-5(ptc)* mRNA decay, while SMG-2 (UPF1) and SMG-4 (UPF3) impact *unc-89(ptc)* mRNA decay. Similar observations were made in mouse *Actb* and *Rela* mutant cells in which siRNA-mediated knock down of SMG6 blocked the transcriptional adaptation response in *Actb* mutant mESCs but had little influence on *Rela* mutant MEFs. Conversely, siRNA-mediated knockdown of UPF1 blocked the transcriptional adaptation response in *Rela* mutant MEFs but not in *Actb* mutant mESCs (El-Brolosy et al., 2019). Consistently, mutant mRNA decay can involve different factors in the same organism (Nickless et al., 2017), possibly due to differential expression of the decay factors. However, we cannot exclude the possibility that SMG6 could function as a decay factor independent of NMD, especially since it has been reported to have NMD-independent cleavage activity (Gehring et al., 2005; Glavan et al., 2006; Huntzinger et al., 2008; Chakrabarti et al., 2014).

Transcriptional adaptation can be triggered by the degradation products of the mutant mRNA (El-Brolosy et al., 2019), which could seed the generation of sRNAs (Mattick and Makunin, 2005; Boivin et al., 2018). We found that factors involved in sRNA maturation and transport, including RRF-3, DCR-1, ERGO-1 and NRDE-3, also regulate transcriptional adaptation. Transcriptional modulation of genes by sRNAs of approximately 20–30 nucleotides in length is a widespread and diverse feature of prokaryotes (Melamed et al., 2019) and eukaryotes (Ambros et al., 2003; Yigit et al., 2006; Hutvagner and Simard, 2008; Portnoy et al., 2011; Castel and Martienssen, 2013; Rechavi and Lev, 2017; Billmyre et al., 2019). Notably, the factors we identified are known to be involved in somatic gene regulation by sRNAs, described as the RRF-3 pathway (Gent et al., 2010). RRF-3 is an RNA-dependent RNA polymerase involved along with the DICER complex in the biogenesis of 26-nucleotide RNAs with 5' bias for guanosine monophosphate (26G-RNAs) (Han et al., 2009; Gent et al., 2010; Vasale et al., 2010). 26G sRNAs associate with the Argonaute protein ERGO-1, which is involved in the further maturation of sRNAs and is required to separate the sRNA duplex (Han et al., 2009; Gent et al., 2010; Fischer et al., 2011). Mature sRNAs interacting with Argonaute proteins can direct post-transcriptional gene silencing (Vasale et al., 2010; Phillips et al., 2012), or be transported into the nucleus (Guang et al., 2008; Buckley et al., 2012; Shirayama et al., 2012). NRDE-3 is another Argonaute protein involved in transporting sRNAs into the nucleus (Guang et al., 2008), and we found that knocking it down, and knocking it out, blocked transcriptional adaptation while not affecting mutant mRNA levels. While sRNAs are best known as repressors of gene expression, they can also function as activators (Li et al., 2006; Janowski et al., 2006; Turunen et al., 2009; Portnoy et al., 2011; Wedeles et al., 2013; Li, 2017), although the underlying mechanisms remain poorly understood (Portnoy et al., 2011). Some of these activating sRNAs interact with Argonaute proteins (Seth et al., 2013), and they can target gene regulatory sequences including promoters. Whether they can also interfere with antisense RNAs, which usually function to repress gene expression (Faghihi and Wahlestedt, 2009; Modarresi et al., 2012), is a hypothesis worth testing given our observations in zebrafish embryos and mouse cell lines (El-Brolosy et al., 2019) as well as the previously suggested role of Argonaute proteins in such a process (Ghanbarian et al., 2017).

The transcriptional adaptation factors identified here came from a candidate screen where we specifically targeted pathways involved in RNA metabolism. With this study, we have established *C. elegans* as a genetic model system to perform unbiased screens to help reveal further mechanisms of transcriptional adaptation, a newly uncovered phenomenon contributing to genetic robustness.

## Materials and methods

**Key resources table:** 

Reagent type (species) or resource	Designation	Source or reference	Identifiers	Additional information
Gene (*Caenorhabditis elegans*)	*act-1*		CELE_T04C12.6	WormBase ID: WBGene00000063
Gene (*Caenorhabditis elegans*)	*act-2*		CELE_T04C12.5	WBGene00000064
Gene (*Caenorhabditis elegans*)	*act-3*		CELE_T04C12.4	WBGene00000065
Gene (*Caenorhabditis elegans*)	*act-4*		CELE_M03F4.2	WBGene00000066
Gene (*Caenorhabditis elegans*)	*act-5*		CELE_T25C8.2	WBGene00000067
Gene (*Caenorhabditis elegans*)	*unc-89*		CELE_C09D1.1	WBGene00006820
Gene (*Caenorhabditis elegans*)	*sax-3*		CELE_ZK377.2	WBGene00004729
Strain, strain background (*C. elegans*)	N2	CGC, Bristol strain		wild type
Strain, strain background (*C. elegans*)	IN2049	MacQueen et al., 2005		*act-5(ptc)*; *dtIs419[act-5+ rol-6(d)]*
Strain, strain background (*C. elegans*)	IN2051	MacQueen et al., 2005		*act-5(Δ1); dtIs419[act-5+ rol-6(d)]*
Strain, strain background (*C. elegans*)	VC971	CGC, Estes et al., 2011		+/mT1; *act-5*(*Δ2*)/*mT1 [dpy-10(e128)]*.
Strain, strain background (*C. elegans*)	CB4043	CGC, Hodgkin et al., 1989		*smg-2(e2008);him-5(e1490)*
Strain, strain background (*C. elegans*)	CB4355	CGC, Pulak and Anderson, 1993		*smg-4(ma116);him-8(e1490)*
Strain, strain background (*C. elegans*)	TR1396	CGC, Pulak and Anderson, 1993		*smg-6(r896)*
Strain, strain background (*C. elegans*)	YY168	CGC, Pavelec et al., 2009		*ergo-1(gg100)*
Strain, strain background (*C. elegans*)	YY158	CGC, Guang et al., 2008		*nrde-3(gg66)*
Strain, strain background (*C. elegans*)	YY13	CGC, Pavelec et al., 2009		*rrf-3(mg373)*
Strain, strain background (*C. elegans*)	DYS0005	This study, crossed IN2049 to N2		*act-5(ptc)*
Strain, strain background (*C. elegans*)	DYS0004	This study, crossed IN2049 to N2		*+/act-5(Δ1)*
Strain, strain background (*C. elegans*)	DYS0012	This study, injected in N2		*Ex[act-5p::RFP]*
Strain, strain background (*C. elegans*)	DYS0014	This study, injected in N2		*Ex[act-3p::RFP]*
Strain, strain background (*C. elegans*)	DYS0015	This study, crossed DYS0014 to DYS0004		*act-5(ptc);Ex[act-3p::RFP]*
Strain, strain background (*C. elegans*)	DYS0042	This study, crossed DYS0012 to DYS0005		*act-5(ptc);Ex[act-5p::RFP]*
Strain, strain background (*C. elegans*)	VC40114	CGC, Million Mutation Project		*unc-89(ptc1)*
Strain, strain background (*C. elegans*)	VC40193	CGC, Million Mutation Project		*unc-89(ptc2)*
Strain, strain background (*C. elegans*)	VC40199	CGC, Million Mutation Project		*unc-89(ptc3)*
Strain, strain background (*C. elegans*)	DYS0028	This study, crossed VC40114 to N2		*unc-89(ptc1)*
Strain, strain background (*C. elegans*)	DYS0030	This study, crossed VC40193 to N2		*unc-89(ptc2)*
Strain, strain background (*C. elegans*)	DYS0031	This study, crossed VC40199 to N2		*unc-89(ptc3)*
Strain, strain background (*C. elegans*)	DYS0037	This study, induced by CRISPR/Cas9		*unc-89(Δ)*
Strain, strain background (*C. elegans*)	DYS0008	This study, crossed DYS0005 to CB4043		*smg-2(e2008); act-5(ptc)*
Strain, strain background (*C. elegans*)	DYS0057	This study, crossed DYS0005 to CB4355		*act-5(ptc); smg-4(ma116)*
Strain, strain background (*C. elegans*)	DYS0047	This study, crossed DYS0028 to CB4355		*unc-89(ptc1); smg-4(ma116)*
Strain, strain background (*C. elegans*)	DYS0048	This study, crossed DYS0030 to CB4355		*unc-89(ptc2); smg-4(ma116)*
Strain, strain background (*C. elegans*)	DYS0050	This study, crossed DYS0031 to CB4355		*unc-89(ptc3); smg-4(ma116)*
Strain, strain background (*C. elegans*)	DYS0053	This study, crossed DYS0028 to TR1396		*unc-89(ptc1); smg-6(r896)*
Strain, strain background (*C. elegans*)	DYS0055	This study, crossed DYS0030 to TR1396		*unc-89(ptc2); smg-6(r896)*
Strain, strain background (*C. elegans*)	DYS0056	This study, crossed DYS0031 to TR1396		*unc-89(ptc3); smg-6(r896)*
Strain, strain background (*C. elegans*)	DYS0010	This study, crossed DYS0005 to YY168		*act-5(ptc); ergo-1(gg100)*
Strain, strain background (*C. elegans*)	DYS0054	This study, crossed DYS0028 to YY168		*unc-89(ptc1); ergo-1(gg100)*
Strain, strain background (*C. elegans*)	DYS0051	This study, crossed DYS0030 to YY168		*unc-89(ptc2); ergo-1(gg100)*
Strain, strain background (*C. elegans*)	DYS0052	This study, crossed DYS0031 to YY168		*unc-89(ptc3); ergo-1(gg100)*
Strain, strain background (*C. elegans*)	DYS0045	This study, crossed DYS0005 to YY158		*act-5(ptc); nrde-3(gg66)*
Strain, strain background (*C. elegans*)	DYS0065	This study, crossed DYS0028 to YY158		*unc-89(ptc1); nrde-3(gg66)*
Strain, strain background (*C. elegans*)	DYS0072	This study, crossed DYS0030 to YY158		*unc-89(ptc2); nrde-3(gg66)*
Strain, strain background (*C. elegans*)	DYS0066	This study, crossed DYS0031 to YY158		*unc-89(ptc3); nrde-3(gg66)*
Strain, strain background (*C. elegans*)	DYS0046	This study, crossed DYS0005 to YY13		*rrf-3(mg373); act-5(ptc)*
Strain, strain background (*C. elegans*)	DYS0070	This study, crossed DYS0028 to YY13		*unc-89(ptc1); rrf-3(mg373)*
Strain, strain background (*C. elegans*)	DYS0062	This study, crossed DYS0030 to YY13		*unc-89(ptc2); rrf-3(mg373)*
Strain, strain background (*C. elegans*)	DYS0063	This study, crossed DYS0031 to YY13		*unc-89(ptc3); rrf-3(mg373)*
Commercial assay or kit	In-Fusion HD Cloning	Clontech	Clontech:639647	
Commercial assay or kit	Superscript III reverse transcriptase	Takara	Cat. No: 18080–044	
Commercial assay or kit	SMARTer RACE cDNA Amplification Kit	Takara	Cat. N. 634860	
Commercial assay or kit	Advantage 2 PCR kit	Takara	Cat. N. 639207	
RNAi construct	mv_C18D11.4	BioScience		*rsp-8*
RNAi construct	sjj2_C18D11.4	BioScience		*rsp-8*
RNAi constructs	mv_C33H5.12	BioScience		*rsp-6*
RNAi constructs	sjj2_C33H5.12	BioScience		*rsp-6*
RNAi constructs	mv_W02B12.3	BioScience		*rsp-1*
RNAi constructs	sjj2_W02B12.3	BioScience		*rsp-1*
RNAi constructs	mv_D2089.1	BioScience		*rsp-7*
RNAi constructs	sjj2_D2089.1	BioScience		*rsp-7*
RNAi constructs	mv_B0464.5	BioScience		*spk-1*
RNAi constructs	sjj2_B0464.5	BioScience		*spk-1*
RNAi constructs	mv_R05D11.6	BioScience		*paxt-1*
RNAi constructs	sjj2_R05D11.6	BioScience		*paxt-1*
RNAi constructs	mv_F43E2.8	BioScience		*hsp-4*
RNAi constructs	sjj2_F43E2.8	BioScience		*hsp-4*
RNAi constructs	sjj2_Y39G8C.1	BioScience		*xrn-1*
RNAi constructs	mv_Y48G8AL.6	BioScience		*smg-2*
RNAi constructs	sjj2_Y48G8AL.6	BioScience		*smg-2*
RNAi constructs	sjj2_F46B6.3	BioScience		*smg-4*
RNAi constructs	mv_Y54F10AL.2	BioScience		*smg-6*
RNAi constructs	sjj2_Y54F10AL.2	BioScience		*smg-6*
RNAi constructs	mv_F26B1.2	BioScience		*hrpk-1*
RNAi constructs	sjj2_F26B1.2	BioScience		*hrpk-1*
RNAi constructs	mv_F26E4.10	BioScience		*drsh-1*
RNAi constructs	sjj2_F26E4.10	BioScience		*drsh-1*
RNAi constructs	mv_T22A3.5	BioScience		*pash-1*
RNAi constructs	sjj2_T22A3.5	BioScience		*pash-1*
RNAi constructs	sjj2_F26A3.8	BioScience		*rrf-1*
RNAi constructs	mv_ R06C7.1	BioScience		*wago-1*
RNAi constructs	sjj2_ R06C7.1	BioScience		*wago-1*
RNAi constructs	mv_F58G1.1	BioScience		*wago-4*
RNAi constructs	sjj2_F58G1.1	BioScience		*wago-4*
RNAi constructs	sjj2_F10B5.7	BioScience		*rrf-3*
RNAi constructs	mv_M88.5	BioScience		*zbp-1*
RNAi constructs	sjj2_M88.5	BioScience		*zbp-1*
RNAi constructs	sjj2_K12H4.8	BioScience		*dcr-1*
RNAi constructs	mv_T20G5.11	BioScience		*rde-4*
RNAi constructs	sjj2_T20G5.11	BioScience		*rde-4*
RNAi constructs	mv_F36H1.2	BioScience		*kdin-1*
RNAi constructs	mv_K12B6.1	BioScience		*sago-1*
RNAi constructs	sjj2_K12B6.1	BioScience		*sago-1*
RNAi constructs	mv_K08H10.7	BioScience		*rde-1*
RNAi constructs	sjj2_K08H10.7	BioScience		*rde-1*
RNAi constructs	sjj2_R09A1.1	BioScience		*ergo-1*
RNAi constructs	mv_R04A9.2	BioScience		*nrde-3 *
RNAi constructs	sjj2_R04A9.2	BioScience		*nrde-3*

### Culture conditions and strains

All wild-type worms were the N2 reference strain. All *C. elegans* strains were kept on 6 cm plates with nematode growth medium agar and fed with a lawn of *E. coli* OP50 grown in 500 μl Luria broth, except for the RNAi mediated knockdown experiments where the worms were fed with *E. coli* expressing the respective double-stranded RNA. Cultures were maintained at 20°C. Also, to minimize the potential for laboratory evolution of the trait, a new culture of the strains was revived annually from frozen stocks. All plates with fungal or bacterial contamination were excluded from the experiments.

### Synchronization of cultures for RNA isolation

Worms from healthy cultures were washed off of plates using M9 buffer and passed through a 41 µm filter (Millipore Cat. No SCNY00040) with vacuum; antibiotics (Ampicillin, Chloramphenicol) were added (50 µg/ml final concentration) to eliminate remaining food bacteria, and the worms were then incubated on a shaker at room temperature for 15 min. Worms were centrifuged at 3000 rpm for 5 min to pellet early larval stage animals. The buffer was aspirated and 1 ml of fresh buffer was added to resuspend the pellet. Samples were confirmed to be primarily L1 and L2 stage larvae by observing two 5 µl samples on a 6 cm nematode growth medium plate. Starving cultures or cultures that had more than one male were excluded from the experiments.

### qPCR analysis

Total RNA from synchronized cultures or manually picked young adults was isolated using TRIzol (ambion by Takara). For reverse transcription (RT), Superscript III reverse transcriptase (Invitrogen, Cat. No: 18080–044) was used following manufacturer's instructions. We used 1–2 μg total RNA for each RT reaction. The qPCR experiments were performed on a CFX Connect Real-Time System (Biorad-Roche Diagnostics) as described previously (El-Brolosy et al., 2019). *cdc-42* and *Y45F10D.4* (*iscu-1*) were used as reference genes as described previously (Hoogewijs et al., 2008), and the Ct values ranged from 12.3 to 28.4 for *cdc-42* and 11.8 to 26 for *Y45F10D.4*. The Ct values for all other genes were aimed to be below 30.

The following primers were used to amplify the cDNA of target genes: *Y45F10D.4* (forward 5’- CGAGAACCCGCGAAATGTCGGA-3’ and reverse 5’- CGGTTGCCAGGGAAGATGAGGC-3’), *cdc-42* (forward 5’-AGCCATTCTGGCCGCTCTCG-3’ and reverse 5’- GCAACCGCTTCTCGTTTGGC-3’), *act-1* (forward 5’-ACGACGAGTCCGGCCCATCC-3’ and reverse 5’-GAAAGCTGGTGGTGACGATGGTT-3’), *act-2* (forward 5’-GCGCAAGTACTCCGTCTGGATCG-3’ and reverse 5’- GGGTGTGAAAATCCGTAAGGCAGA-3’), *act-3* (forward 5’-AAGCTCTTCGCCTTACCATTTTCTC-3’ and reverse 5’-ACAGAGCAAATTGTAGTGGGGTCTTC-3’), *act-4* (forward 5’-AGAGGCTCTCTTCCAGCCATCCTTC-3’ and reverse 5’-TGATCTTGATCTTCATGGTGGATGG-3’), *act-5* (forward 5’- AAGTGCGATGTCGACATCAGAAAG-3’ and reverse 5’- TAATCTTGATCTTCATTGTGCTTGG-3’), *act-5d* (forward 5’- AAGTGCGATGTCGACATCAGAAAG-3’ and reverse 5’- TAATCTTGATCTTCATTGTGCTCCGG-3’), *unc-89* (forward 5’-AAGGCTGAACTTGTCATCGAAGGAG-3’ and reverse 5’-TCATCTCCACAACATTACCCTCGTG-3’), *sax-3* (forward 5’-TGCCGTTTGTCCCGTAACAACTATG-3’ and reverse 5’-ATCTTCTGAAGCTGACGGGGAGAAC-3’), *act-3* pre-mRNA (forward 5’-TTTTTCAGAACCATGAAGATCA-3’ and reverse 5’-GAAAATGGTAAGGCGAAGAGC-3’), *sax-3* pre-mRNA (forward 5’-TGTAAACCGCACTGCACAAT-3’ and reverse 5’-TCCACCAAGAGCCTGAAAAC-3’). PCR efficiency was determined using external standards on plasmid mini-preparation of cloned PCR products. Expression levels were analyzed by basic relative quantification. qPCR data are based on three biological replicates and three technical replicates for each biological replicate.

### Rapid amplification of cDNA ends (RACE)

Total RNA from manually picked young wild-type and *act-5(Δ2)* mutant adults was isolated using TRIzol (ambion by Takara). 5’ and 3’ RACE ready cDNA was synthesized by reverse transcription PCR using a SMARTer RACE cDNA Amplification Kit following manufacturer's protocol (Cat. N. 634860, Takara). PCRs were performed using an Advantage 2 PCR kit (Cat. N. 639207, Takara). The following gene-specific primers and nested gene-specific primers were used to amplify 3’ and 5’ cDNA ends: act5GSP2 (5’-ACCACCGGAATCGTTTTGGACACCGGAG-3’), act5NGSP2 (5’-GAAGGATATGCCCTCCCACATGCCATCC-3’), act5GSP1 (5'-AAAAATCAGCTTAGAAGCACTTTCGGTG-3’), act5NGSP1 (5’-TCGATGGGCCGGACTCGTCGTACTCCTG-3’), unc89GSP2 (5’-TTTGGTACCATTTGTATAGAGGCGAGTG-3’), unc89NGSP2 (5’-TTCTGAACTGGACAAATCTTGCTTTTCG-3), unc89N1GSP2 (5’- ACTTTCCAGTATCTCCTGGATGTTGCTTC-3’), and unc89N2GSP2 (5’- TTTGAATACTTTTTGATGAACCGTGTGC-3’). RACE experiment revealed an isoform with an alternative start which is present only in *act-5(Δ2)* mutants (Figure 1—figure supplement 1A). This new isoform is not affected by the large deletion, and thus the corresponding mRNA is not degraded (Figure 1A).

### Plasmid construction and genetic transformation

To study the expression of *act-5*, we generated a reporter construct with an *act-5* promoter region (2.5 kb from III:13606066 to 13608569) fused to turboRFP in a pUC19 vector. Similarly, a pUC19 vector containing turboRFP was fused with an *act-3* promoter region (4.5 kb from V:11073234 to 11077791). The germ line of wild-type animals was injected with the generated plasmids (10 ng ul^−1^). The transgenic lines were subsequently crossed with *act-5(ptc)* mutants to transfer the extrachromosomal array to the mutant background.

### Confocal microscopy

A Zeiss LSM 700 confocal microscope was used to image adult worms.

### RNA interference mediated knockdown

RNAi was performed by feeding double-stranded RNA-expressing bacteria at 25°C from the early larval stage through adulthood (60–75 hr) as previously described (Fraser et al., 2000). For the genes whose knockdown from an early larval stage caused lethality or sterility, we started the RNAi treatment at later stages (L4, adult). Also, for some clones (mv_R05D11.6, sjj2_R05D11.6, sjj2_Y39G8C.1), we diluted the double-stranded RNA-expressing bacteria with empty vector (L4440)-containing bacteria, in order to obtain milder effects. RNAi constructs were obtained from available libraries (Source BioScience) and verified by sequencing. RNAi clones used in this study are listed in the key resources table. 

### CRISPR/Cas9 induced mutations

To generate the CRISPR/Cas9-induced *unc-89* deletion allele (*bns7000*), two sgRNAs (final concentration 4 uM each) were injected with Cas9 protein (0.35 ug/ul), and a *dpy-10* sgRNA (2.5 uM) was used as a co-injection marker along with a repair oligo (PSdpy-10-PS; 0.73 uM) (Dickinson and Goldstein, 2016).

sgRNA1: 5′-GGTAGTTAGCGACCCCATGAGGG-3′.
sgRNA2: 5′-ACAGACTGGTAAACAAACGAGGG-3′

The following primers were used for genotyping: dunc-89–1 forward (5’-ATACCACCACATGTCTCTTC-3’), dunc-89–2 forward (5’-GCTAAAAGTCAGAGTTCCAC-3’), dunc-89–3 reverse (5’- GGATGGGTTTACATAAAAT-3’), dunc-89–4 reverse (5’-TGAAAAAGAAACAACAAAA-3’), dunc-89–5 forward (5’-TAACAAAAAGCTCAAAATG-3’), dunc-89–6 reverse (5’-GGATAGATTTCTGTTGGAGA-3’). The external primers flank a 19612 bp region in wild types and amplify a 3601 bp fragment in *bns7000* mutants. The internal primers with different combinations amplify 500-2600 bp products in wild types.

### Double mutant analysis

All the double mutants exhibited gene expression levels as in the RNAi treated animals with one exception. *act-5(ptc); nrde-3* double mutants exhibited *act-5* mRNA levels as in the RNAi experiments but also some upregulation of the adapting gene, unlike what was observed in the RNAi experiments (Figures 4 and 5, Figure 5—figure supplement 1A and B). One possible explanation is related to an alternative start site of *nrde-3* (Tourasse et al., 2017) which might be used only in some tissues and thus could lead to some protein function in the allele used in our study.

### Statistical evaluation

To calculate the significance of the differences for the expression data, we performed two-tailed Student’s t-test. Mean ± SEM is indicated in graphs. All statistical analyses were implemented in the program Statistica v. 9. Graphs were generated in Prism5.

### Gene structure visualization

The *act-5* and *unc-89* loci were visualized using the GSDS gene structure visualization tool (Guo et al., 2007).

## Data Availability

All data generated or analyzed during this study are included in the manuscript and supporting files.
