# Supplementary material for: Transcriptional adaptation in Caenorhabditis elegans
Source: eLife. 2020 Jan 17;9:e50014. doi: 10.7554/eLife.50014 (PMC6968918; doi:10.7554/eLife.50014)
Supplement: Figure 1—source data 1. [file elife-50014-fig1-data1.pdf]

| target | *act-5* | | | | *act-3* | | | |
|---|---|---|---|---|---|---|---|---|
| sample | WT | *ptc* | *Δ1* | *Δ2* | WT | *ptc* | *Δ1* | *Δ2* |
| dCt value | -1.6 | 0.1 | -1.7 | -2.7 | -5.9 | -7 | -5.7 | -5.7 |

**Figure 1-source data 1.**
