# Supplementary material for: Transcriptional adaptation in Caenorhabditis elegans
Source: eLife. 2020 Jan 17;9:e50014. doi: 10.7554/eLife.50014 (PMC6968918; doi:10.7554/eLife.50014)
Supplement: Figure 1—source data 2. [file elife-50014-fig1-data2.pdf]

target	*act-1*		*act-2*		*act-4*	
sample	WT	*ptc*	WT	*ptc*	WT	*ptc*
dCt value	1.8	1.6	0.86	0.79	-1.7	0.2

Figure 1- source data 2.
