# Supplementary material for: Transcriptional adaptation in Caenorhabditis elegans
Source: eLife. 2020 Jan 17;9:e50014. doi: 10.7554/eLife.50014 (PMC6968918; doi:10.7554/eLife.50014)
Supplement: Figure 3—source data 1. [file elife-50014-fig3-data1.pdf]

Gene	methods
F21C10.7	Panther, GeneACeDB-Compara, WormBase-Compara
F32D8.1	TreeFam
R06A10.4	TreeFam
cmk-1	TreeFam
dapk-1	TreeFam
dim-1	Panther
him-4	GeneACeDB-Compara, WormBase-Compara
igcm-2	GeneACeDB-Compara
igdb-1	Panther
igdb-2	Panther
ketn-1	Panther, GeneACeDB-Compara, WormBase-Compara
mlck-1	TreeFam
rig-1	Panther
sax-3	GeneACeDB-Compara, WormBase-Compara
sax-7	Panther
unc-22	Panther, GeneACeDB-Compara
unc-89	WormBase-Compara

Figure 3-source data 1
