# Supplementary material for: Transcriptional adaptation in Caenorhabditis elegans
Source: eLife. 2020 Jan 17;9:e50014. doi: 10.7554/eLife.50014 (PMC6968918; doi:10.7554/eLife.50014)
Supplement: Figure 3—source data 2. [file elife-50014-fig3-data2.pdf]

target	*unc-89*					*sax-3*				
sample	WT	*ptc1*	*ptc2*	*ptc3*	*Δ*	WT	*ptc1*	*ptc2*	*ptc3*	*Δ*
dCt value	-2.7	-2.1	-2.1	-1.8	-28.3	1.6	0.4	1.4	1.4	1.7

Figure 3-source data 2.
