# Supplementary material for: Transcriptional adaptation in Caenorhabditis elegans
Source: eLife. 2020 Jan 17;9:e50014. doi: 10.7554/eLife.50014 (PMC6968918; doi:10.7554/eLife.50014)
Supplement: Figure 3—source data 3. — ‘-’ indicates the absence of the PTC from the isoform. [file elife-50014-fig3-data3.pdf]

allele	*ptc1*	*ptc2*	*ptc3*	*ptc1*	*ptc1*	*ptc3*
isoform	distance from the PTC to the exon-intron junction (bp)			distance from the PTC to the stop codon (bp)		
a	283	133	-	666	234	-
b	283	133	212	5013	4581	4039
c	-	-	212	-	-	4011
d	-	-	-	-	-	-
e	283	133	-	666	234	-
f	283	133	212	5013	4581	4011
g	283	133	212	5013	4581	4011
h	283	133	212	5013	4581	4011
i	283	133	212	5013	4581	4011
j	283	133	212	5013	4581	4011
k	283	133	212	5013	4581	4011
l	283	133	-	666	234	-
m	283	133	212	5013	4581	4011
n	283	133	212	5013	4581	4011
o	283	133	-	666	234	-
p	283	133	212	5013	4581	4011

Figure 3-source data 3.
